# Boron Nitride Nanotubes as Filler for Resin-Based Dental Sealants

**DOI:** 10.1038/s41598-019-44246-8

**Published:** 2019-05-22

**Authors:** Fabio Rocha Bohns, Felipe Weidenbach Degrazia, Gabriela de Souza Balbinot, Vicente Castelo Branco Leitune, Susana Maria Werner Samuel, Maria Angeles García-Esparza, Salvatore Sauro, Fabricio Mezzomo Collares

**Affiliations:** 1 Dental Materials Laboratory, School of Dentistry. Universidade Federal do Rio Grande do Sul, Porto Alegre, Brazil; 2 Department of Pharmacy, Universidad Cardenal Herrera-CEU Universities, Elche (Alicante), Spain; 3 Departamento de Odontologia, Facultad de Ciencias de la Salud, Universidad CEU Cardenal Herrera, Alfara del Patriarca (Valencia), Spain

**Keywords:** Biomineralization, Health occupations

## Abstract

The aim of this study was to evaluate the influence of boron-nitride nanotubes (BNNTs) on the properties of resin-based light-curing dental sealants (RBSs) when incorporated at different concentration. RBSs were formulated using methacrylate monomers (90 wt.% TEGDMA, 10 wt.% Bis-GMA). BNNTs were added to the resin blend at 0.1 wt.% and 0.2 wt.%. A Control group without filler was also designed. Degree of conversion, ultimate tensile strength, contact angle, surface free energy, surface roughness and color of the RBSs were evaluated for the tested materials. Their cytotoxicity and mineral deposition ability (Bioactivity) were also assessed. A suitable degree of conversion, no effect in mechanical properties and no cytotoxic effect was observed for the experimental materials. Moreover, the surface free energy and the surface roughness decreased with the addition of BNNTs. While the color analysis showed no difference between specimens containing BNNTs and the control group. Mineral deposition occurred in all specimens containing BNNTs after 7d. In conclusion, the incorporation of BNNTs may provide bioactivity to resin-based dental sealants and reduce their surface free energy.

## Introduction

Resin-based dental sealants (RBSs) are recommended for microinvasive treatments for caries lesions^1^. This treatment aims to physically seal the dental tissues in order to avoid biofilm accumulation in those areas at potentially risk of caries, such as occlusal and proximal surfaces. This results in the prevention of enamel-dentin demineralization and the formation of carious lesions; any preventive approach to control the progression of disease may reduce the need for invasive treatments^2^. Moreover, pits and fissures sealing offers reliable protection against caries, which reduces the incidence of such a disease up to 18,92% in permanent molars in 5 to 10 years old children^3^. The effectiveness of resin-based sealants is well established^4,5^ as a cost-effective treatment for controlling caries lesions^5–7^.

However, the efficiency of RBSs in caries control would be enhanced if such material had some bioactivity and antibacterial properties to reduce biofilm growth^8^. Indeed, such properties would contribute to the healing of early caries lesions and increase the anti-caries activity of RBS’s, respectively. Moreover, fillers with remineralization and antibiofilm properties may also offer the possibility to protect dental hard tissues from acidic challenges via a buffering process^9^. Fluoride has been extensively used in dental sealants as an active anti-caries agent, but its benefits remain still unclear^9^. Moreover, the release of fluoride from resin-based materials may rapidly decrease, so reducing its antibacterial effect over time^10^.

Boron-nitride nanotubes (BNNTs) are nanomaterials analogous to carbon nanotubes^11^. Due to their nanometric size, high surface/volume ratio, surface functionality, high elastic modulus, biocompatibility^12^ hydrophobicity, chemical inertness and susceptibility to precipitation in water solutions, BNNTs have gained great attention in biomedical science^13^. The apatite formation ability of BNNTs in physiological solutions has been advocated^14^, even when these were used as filler for resin dental adhesives^15^. The precipitation of minerals promoted by BNNTs might contribute to the therapeutic effects of RBS in reducing the risk of caries in kids and adults.

The addition of this nanometric filler may affect the monomer conversion, the mechanical properties, the wetting ability, the surface properties, the color and the biocompatibility of these materials. Thus, the aim of this study was to investigate the influence of BNNTs addition to an experimental methacrylate-based sealant. This objective was accomplished through the evaluation of the degree of conversion, contact angle, surface free energy, surface roughness and color of the tested RBSs doped with BNNTs, which were compared to a filler-free control RBS. Cytotoxicity assessment and mineral deposition (bioactivity) at 7, 14 and 28 days of immersion in artificial saliva (AS) were also evaluated.

## Results

**Figure 1:**
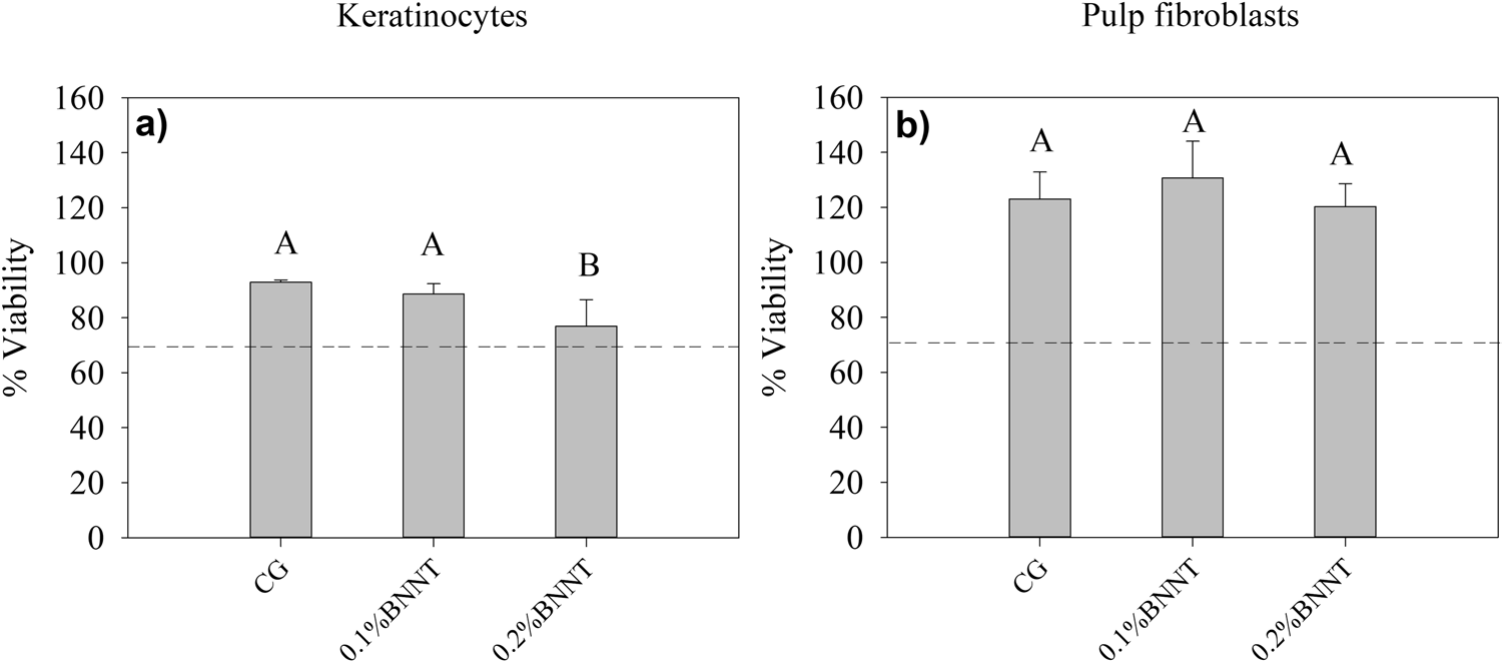
Cell viability (%) of experimental resin-based sealants against keratinocytes (Fig. 2A) and pulp fibroblasts (Fig. 2B) cell lines. The uppercase letters indicate significant statistical difference within each cell type.

**Figure 2:**
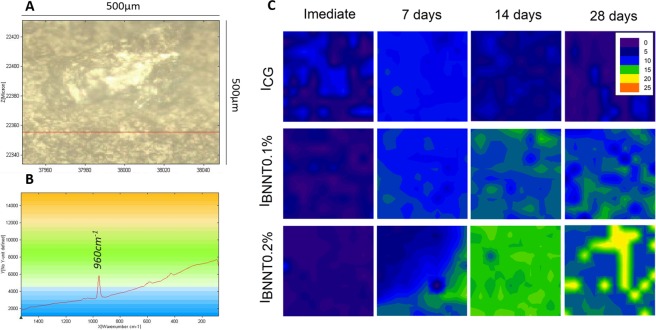
Raman analysis of mineral deposition after 7, 14 and 21 days after immersion of samples in artificial saliva. (**A**) Representative image of the analyzed area (500 µm × 500 µm). (**B**) Representative Raman spectrum showing the PO_4_^3−^ peak (960 cm^−1^). (**C**) Images obtained after the integration of the absorbance peak of 960 cm^−1^ on micro Raman spectrometer. The increase in phosphate peak (960 cm^−1^) is represented by the different colors in the images increasing from blue to orange as shown in the legend. Mineral deposits were found after 7 days of immersion in artificial saliva for the groups containing BNNTs. Increase of mineral deposition was found on the surface of experimental sealants containing higher amount of BNNTs concentration.

**Figure 3:**
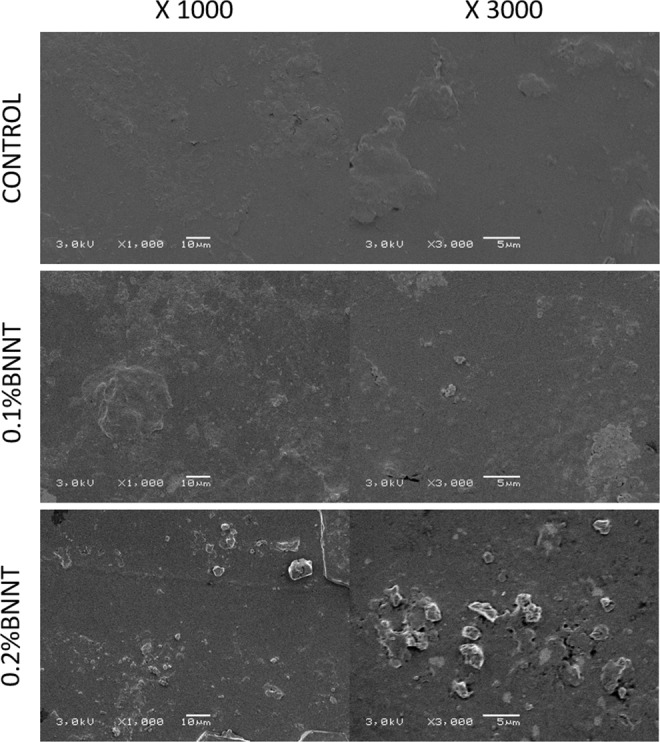
SEM images of the surface of specimens after immersion in artificial saliva for 28 days. Mineral deposition was found over the surface of specimens containing BNNTs.

The DC of the tested resin-based sealants is shown in Table 1. No significant statistical difference was found between the DC values obtained with the different experimental resin-based sealants (p > 0.05). The addition of BNNTs did not influence the UTS as shown in Table 1. The results of SFE and contact angles for water and α-bromonaphthalene on enamel specimens treated with the tested RBSs are depicted in Table 2. A decrease in SFE was found in RBBs containing 0.1 wt.% (52.36 ± 3.48) and 0.2 wt.% (52.42 ± 1.61) of BNNTs (p < 0.05). The mean and standard deviation values for surface energy of experimental RBS in sound and demineralized enamel surface are presented in Table 2. No significant difference was observed between the tested RBS applied in the same substrate (Sound or demineralized) (p > 0.05). However, significantly lower angle values were obtained in demineralized enamel when using the BNNT-containing RBSs (p < 0.05) compared to the values obtained in sound enamel. Surface roughness was higher in demineralized enamel as well as in all surfaces treated with I_GC_ RBS (p < 0.05; Table 1). No significant difference was found between sound enamel and enamel surfaces treated with I_0.1%BNNT_ and I_0.2%BNNT_ RBSs (p < 0.05). Colorimetric results of RBS-sealed enamel calculated for both sound (ΔE1) and demineralized (ΔE2) are represented in Table 1. A significant difference was found between sound and demineralized enamel for all groups (p < 0.05). Cytotoxic results for keratinocytes (a) and dental pulp fibroblasts (b) are shown in Fig. 1. The rate of viability for both cell lines cells remained above 75% in all materials containing BNNT. However, reduced cell viability was observed for keratinocytes in the group containing 0.2 wt.% BNNTs (p < 0.05). No statistical difference was observed for pulp fibroblasts cells (p > 0.05). Mineral deposition was found on specimens containing 0.1 wt.% and 0.2 wt.% of BNNTs after immersion in artificial saliva for 14 and 7 days, respectively (Fig. 2). Scanning electron microscopy images of the specimens after 28 days in soaking media show mineral precipitation over BNNT-containing groups (Fig. 3).

**Table 1:** Results of the degree of conversion, ΔE calculated using CIELab for sound (ΔE1) and demineralized (ΔE2) enamel and surface roughness represented as means ± SD for each group.

Groups	DC (%)	Sound (ΔE1)	Demineralized (ΔE2)	Surface Roughness (µm)	Ultimate Tensile Strenght (MPa)
Sound	—	—	—	0.86 (±0.28)^A^	—
Demineralized	—	—	—	3.06 (±1.00)^B^	—
I_CG_	73.01(±5.57)^A^	4.04(±2.51)^Aa^	13.01 (±4.09)^Ab^	2.36 (±0.58)^B^	19,46 (±4,78)^A^
I_0.1%BNNT_	72.66(±2.25)^A^	4.34 (±1.51)^Aa^	12.83 (±2.17)^Ab^	2.42 (±0.60)^B^	18,66 (±3,16)^A^
I_0.2%BNNT_	71.13(±2.95)^A^	4.09 (±1.14)^Aa^	12.48 (±4.17)^Ab^	2.44 (±0.49)^B^	17,99 (±2,37)^A^

Different lowercase letter indicates statistical difference in the same row (p < 0.05). Different uppercase letter indicates statistical difference in the same column (p < 0.05).

— Not evaluated.

**Table 2:** Values represented as means ± SD of the contact angles of distilled water and α-Br droplets over sealed enamel specimens’ surface and related surface free energy.

Groups	Contact Angle [θ]		SFE [mN/m]
	Water	α-Br	
I_CG_	45.67 (±12.40)^A^	17.60 (±5.51)^A^	60.84 (±4.74)^B^
I_0.1%BNNT_	60.65 (±11.57)^A^	13.67 (±5.58)^A^	52.36 (±3.48)^A^
I_0.2%BNNT_	55.59 (±4.68)^A^	21.71 (±5.03)^A^	52.42 (±1.61)^A^

Different uppercase letter indicates statistical difference between groups (p < 0.05).

## Discussion

Several properties of resin-based sealants can be influenced by the incorporation of BNNTs nanofillers. In particular, such fillers can influence the therapeutic bioactivity of RBSs; this may potentially prevent and/or reduce the progression of caries lesions when applied in a clinical scenario. Indeed, the incorporation of BNNTs within the composition of resin-based sealants may result in a suitable method to generate new innovative remineralising materials for application in those patients with high risk of caries^9^. The results of this study showed that the presence of BNNTs in the tested experimental RBSs promoted mineral deposition between a period of 7 and 14 days of immersion in AS.

On the other hand, the addition of nanofillers had no negative effect on the physicochemical properties of the experimental BNNT-RBSs; the only exception was for a decrease in SFE observed in enamel surface after treatment with 0.2 wt.% BNNTs containing RBS. Although low BNNT filler concentrations were incorporated (0.1 and 0.2 wt%) into the experimental RBSs, a great volume of particles was added to the resin blend due to the high surface area of the nanotubes^16^; this may justify the SFE results attained in this current study. The BNNTs used in these study presents a high aspect ratio due to the length of nanotubes, which along with the nanosized diameter, contributes to the increased surface area^17^. An adequate dispersion of such fillers within the polymeric matrix may contribute to the adequate formation of polymeric chains^18^; in the present study, this was guaranteed by the addition of 10% Bis-GMA. Indeed, the aromatic groups present in Bis-GMA may have increased the interaction between the polymeric matrix and the fillers. This is because BNNTs’ sidewalls display an affinity to chemical compounds with aromatic groups due to the π-stacking mechanism or hydrophobic interactions^19,20^. For this reason, BNNTs were mixed with Bis-GMA prior to the addition of TEGDMA during the preparation of the experimental RBS tested in this study. This allowed adequate dispersion of the nanotubes within the polymer matrix, which likely avoided issues related to “poor” monomer conversion (Table 1). One could expect that the volume of BNNT particles or the addition of Bis-GMA in the polymeric blend would affect the wettability of sealants. However, this was maintained for all the experimental materials; this can be observed in the contact angle results (Table 3).

**Table 3:** Values represented as means ± SD of the contact angles of resin sealants in sound and demineralized enamel surfaces.

Groups	Contact Angle [θ]	
	Sound	Demineralized
I_CG_	24.70 (±3.99)^Aa^	11.56 (±4.25)^Ab^
I_0.1%BNNT_	31.73 (±6.36)^Aa^	9.45 ± (1.60)^Ab^
I_0.2%BNNT_	30.57 (±10.95)^Aa^	10.8 ± (3.66)^Ab^

Different lowercase letter indicates statistical difference between different enamel surfaces (p < 0.05).

The interaction of polymer and filler, along with an adequate polymerization reaction may increase material stability and reduce the release of polymeric compounds. The adequate conversion of monomers and the dispersion of particles in the matrix are related to adequate mechanical properties and, as shown in Table 1, BNNTs addition showed no effect in the ultimate tensile strength of developed RBSs. Besides, the adequate formation of the polymeric matrix reduces the leaching of unreacted monomers, especially TEGDMA which is one of the main leachable monomers in dental resin blends^21^. The lipophilic nature of this material may be associated with cytotoxic effects, which easily diffuse as unreacted monomer through cellular membrane^22^. The high DC found for all tested RBSs implies low release of monomers and, thus, the low effect in cell viability (Fig. 1). The HaCaT cell line may be more sensitive than primary fibroblasts to TEGDMA-rich polymeric blends containing BNNTs, as all concentrations reached levels of viability over 80%. This was used for testing the cytotoxicity of products that may be in contact with connective and epithelial tissue. A cytotoxic effect of BNNTs was previously advocated due to an asbestos-like shape^23^, but no cytotoxic effect was observed when different cell lines were tested^12,24^. In this study, no relevant cytotoxic was observed in the tested RBSs having a concentration of BNNT between 0.1% and 0.2%.

However, it is important to consider that the addition of fillers to dental resin blends may cause important color alteration, which can affect the aesthetic of the treated teeth. It is known that polymeric composites containing carbon nanotubes can present a grey-black aspect^25^, although BNNTs are analogous to carbon nanotubes^11^ and they are usually white-grey in color. To assess the influence of BNNTs addition on the esthetic properties of our experimental sealants, the color of sealed bovine enamel was evaluated and compared to sound (ΔE1) and demineralized (ΔE2) baseline values. Regarding the ΔE means, the color of sealed enamel remained unchanged, with or without nanotubes, in both substrates, while low values of ΔE1 in comparison to ΔE2 were obtained. The significant decreasing of L* and increasing in b* values of resin sealants can make the restoration more similar to the color of sound enamel. The higher the ΔE, the greater the difference in the tested parameters and the perception of color alteration^26^. The mean tolerance for color acceptability in 95% of observers is 4.0 ΔE. Considering such a value for a *in vivo* scenario, the results of ΔE1 obtained in this study can be considered within the limits of tolerance. Thus, the white-grey color of BNNTs seems to have no influence on the color of RBSs and the materials tested in this study had the ability to mask the white aspect of demineralized enamel^27^. As color remained similar to the sound enamel after sealing treatment, it is possible to use this material in aesthetic areas without compromising aesthetics.

Hydrophobicity is a remarkable and well-documented property of BNNTs. In this study, the contact angle values were acceptable although the addition of nanotubes. No change was observed in the contact angle of both water or α-bromonaphthalene when applied on enamel treated with the experimental RBSs. Further, the RBS containing BNNT showed no statistical difference in the contact angles of the material when dropped onto demineralized and sound enamel (Table 3). The experimental RBS tested in this study interacted adequately with both sound and demineralized enamel. This latter situation may be related to the adhesion of resin-based sealants, the longevity of the treatment^5,9,28^ and the extent of the preventive effect. It is well known that high-viscosity resin-based materials may be less able to penetrate into occlusal pits and fissures^29^ and this may affect the micromechanical interaction between the tooth and the resin sealant interface^30^. The low-viscosity of the produced polymeric blend may contribute to this interaction and the adequate wettability found for all groups may contribute to an increased adhesion over time. Both the loss of resin sealants, as well as their degradation, are associated with high-risk of caries^5,31^.

The lower surface energy found for BNNTs containing RBSs may be associated with a possible reduced protein and bacterial attachment to substrates^32^. This reduction found for SFE may enlighten a potential anti-adhesion property of the material, as the interaction between bacteria and substrate was shown to decrease with the decrease of surface energy^33^. This could reduce the risk of caries around an already sealed zone, especially as the surface roughness of sealed enamel was significantly different from the sound tissue for control and experimental groups (Table 1). Rough surfaces may increase the adhesion of bacteria due to the increased surface area available for biofilm formation^34^, which usually occur in cavitated and non-cavitated early enamel lesions. These are plaque retaining sites and, in this situation, conventional home hygiene procedures may not be adequate to reduce the biofilm and the risk for caries. Although results of sealed surfaces are not comparable to sound enamel, the addition of BNNTs caused no significant increase in surface roughness when compared to filler-free RBS.

The addition of particles with remineralization potential aims to improve the performance of the sealant-enamel interface against the acidic challenge caused by oral bacteria. The apatite formation ability of BNNTs after 7, 14 and 28 days once soaked in simulated body fluid was already reported in previous study^14,35^ performed on surfaces of dental adhesives containing BNNTs^15^. The current study showed an increase of the intensity of phosphate peaks (≈960 cm^−1^) in the specimens containing BNNTs (at 7 to 28 days in artificial saliva). The potential of artificial saliva to nucleate Ca-P minerals is already known^36^ and occurs similar to simulated body fluid due to the high ion concentration in the solution. At first, the specimen is covered with an amorphous apatite layer, and, in the second stage, crystalline needle growth is observed as the ion concentration of the soaking media decreases^14^. This may explain the homogeneous deposition over the specimen containing 0.2 wt.% BNNTs at 14 days in artificial saliva, as seen in Fig. 3. No needle-like apatite structure was found over the specimens by SEM, but amorphous apatite was found for the two concentrations after 28 days. This potential bottom-up mineralization that occurred on the specimens containing BNNTs could be a useful mechanism for remineralizing the adjacent tissue, which may enhance performance against recurrent caries.

Innovative strategies for prevention as well as non-invasive treatments to arrest the progression for enamel caries lesions are of major interest nowadays in modern dentistry^37^. The addition of BNNTs at 0.1 wt.% and 0.2 wt.% to low-viscosity polymeric blends may represent a suitable method to create resin-based materials with reduced surface free energy able to promote remineralization of surrounding dental hard tissues (e.g. dentin). These characteristics, combined with an adequate degree of monomer conversion and absence of cytotoxic effects, makes BNNTs-containing resin-based sealants ready for clinical investigation in minimally-invasive procedures to control and arrest caries lesions. The BNNT-containing RBSs can have the ability to reduce the adhesion of bacteria and to promote healing processes of dental hard tissues via mineral deposition. However, *in vivo* studies are necessary to confirm such observations generated in the current study.

## Materials and Methods

### Experimental sealants preparation

An experimental resin blend was prepared by mixing 90 wt% of TEGDMA, 10 wt% of bisphenol A-glycidyl methacrylate (Bis-GMA), 1 mol% camphorquinone (CQ) and 1 mol% of ethyl 4-dimethylaminebenzoate (EDAB), all obtained from Sigma Aldrich (Sigma Aldrich Co.; Jurubatuba, São Paulo, SP, Brazil); this was designed as control sealant (no addition of nanotubes; S_CG_). Hexagonal BNNTs (BNNT, LLC, Newport News, VA, USA) with an average tube length of 200 µm and surface area of 212 m^2^/g were added at two different concentrations of 0.1 wt.% or 0.2 wt.% to create the two experimental sealers (S_BNNT0.1%_ and S_BNNT0.2%_, respectively). To achieve complete homogeneity of the sealants, BNNTs were first ultrasonicated with Bis-GMA and next TEGDMA was added to the mixture, followed by CQ and EDAB with a final ultrasonication in darkness for 480 s^38^.

### Degree of conversion

**Figure 4:**
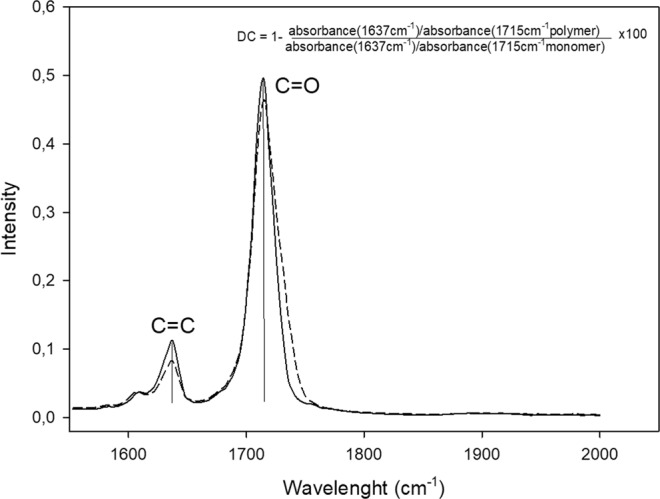
Representative FTIR spectrum with the peaks used to measure the degree of conversion. The C=O and C=C peaks were used to the calculation according to the formula.

The degree of conversion (DC) of the developed sealants was evaluated in a Fourier-transform infrared spectrophotometer (FT-IR Vertex 70, Bruker Optics, Ettlingen, Germany) equipped with an attenuated total reflectance crystal (Platinum ATR-QL, Bruker Optics, Ettlingen, Germany). A small drop of each sealant was poured into a polyvinylsiloxane mould (n = 3), and spectra were assessed prior to and after photo-curing for 40 s with a 1200 mW/cm^2^ (Radii Cal; SDI, Australia). DC was calculated as shown in Fig. 4, using the intensities of the aliphatic C=C peak at 1637 cm^−1^ against an internal standard carbonyl group C=O peak at 1715 cm^−2^; this was done in accordance to previous studies that assessed DC of rich TEGDMA blends^39^.

### Ultimate tensile strength

The ultimate tensile strength (UTS) was evaluated with hourglass-shaped samples (n = 5). The samples measuring 8 mm long, 2 mm wide, 1 mm thick, and cross-sectional area of 1 mm^2^ were produced in a metallic matrix with a polyester strip at the bottom and at the top of the matrix. Samples were photoactivated for 40 s in each side and stored for 24 h at 37 °C before testing. The ultimate tensile strength test was performed in a mechanical testing machine (Shimadzu EZ-SX, Shimadzu Corp., Kyoto, Japan) at a crosshead speed of 1 mm/min until rupture of the specimen.

### Contact angle and surface free energy

The contact angle was evaluated by sessile drop method using a camera-based goniometer. One drop (1 µl) of each sealant was placed on sound and demineralized enamel blocks (n = 5). Enamel blocks were prepared from extracted bovine permanent incisors, which were stored in distilled water prior the use. The labial surface of each tooth was ground flat with silicon-carbide sandpaper (500 grit) under water cooling. Afterwards, the flat surfaces were polished in a polishing machine under water cooling with felt disc saturated with alumina suspension. Prior to demineralization, each enamel surface was partially covered with acid-resistant nail varnish, leaving an open-window area (5 mm × 5 mm). Specimens were demineralized with an acidic solution (pH 4.4) as described in a previous study^40^. The solution was maintained at 37 °C for 72 h and the pH was measured twice a day. In case of a change in pH, the solution was replaced with a fresh one.

As per clinical application, the enamel surface of each specimen was etched with 37% phosphoric acid for 30 s and rinsed with distilled water for 30 s. Fifteen teeth were used, and the three different experimental RBSs were applied (n = 5). Surfaces were air dried, and the experimental sealants were gently applied with a microbrush for 20 s and air-dried for 2 s. Subsequently, the specimens treated with the tested sealers were posterior light-cured (Radii Cal; SDI, Australia) for 20 s.

After 10 s from the initial contact between the droplet and the specimen (θ), an image was recorded and analyzed with OneAttension software (Biolin Scientific, Stockholm, Sweden). Contact angles of each resin were calculated by the mean between five measurements. For surface free energy (SFE) analysis, droplets of 1 µL of distilled water (polar liquid) and α-bromonaphthalene (dispersive liquid) were placed on the surface of sealed enamel blocks (n = 5). OWRK/Fowkes method (in mN/m) was used for SFE calculus purposes, following the formula

$${\gamma }_{ls}={\sigma }_{l}+{\sigma }_{s}-2(\sqrt{{\sigma }_{l}^{D}\cdot {\sigma }_{s}^{D}}+\sqrt{{\sigma }_{l}^{P}\cdot {\sigma }_{s}^{P}}),$$

where γ_ls_ is the interfacial tension, σ_l_ is the liquid surface tension, σ_s_ is the solid surface tension, σ^D^ is the surface tension of the dispersive part and σ^P^ is the surface tension of the polar part.

### Surface roughness

Surface roughness test was carried out using a contact-type profilometer (SJ-201; Mitutoyo, Santo Amaro, SP, Brazil). Sound, demineralized and sealed enamel blocks measuring 5 mm in length, 5 mm in width and 2 mm in height (n = 6) were used. Enamel blocks were prepared as described in section 2.4. Each specimen was put on a flat plane, and the profilometer needle was placed on the enamel block aligned to its surface. The values obtained were calculated by the arithmetic mean (Ra) of three scanning lines at different locations of each specimen; 0.8 mm was used as cut-off length.

### Color assessment

Color measurements were performed by a reflectance spectrophotometer (CARY 5000 UV-Vis-NIR; Agilent, Santa Clara, US) equipped with a DRA-1800 integrating sphere. Enamel blocks with sound, demineralized and sealed enamel were positioned over a standard white background. A dark mask containing an opening measuring 4 mm in diameter was placed over the tooth surface to set the limits of analysis. The color of each specimen was measured and quantified in terms of three coordinate values (L*, a* and b*) (n = 5). The color of the sound, demineralized and sealed enamel was assessed and recorded (M1, M2 and M3, respectively) under standardized conditions according to CIE L*a*b* system. The differences on the parameters obtained (ΔL*, Δa* and Δb*) were calculated by M1, M2 and M3. Comparisons between sealed enamel block color and sound enamel (M1 × M3) and demineralized enamel (M2 × M3) were calculated. The overall changes in color impression (ΔE) were calculated using the following formulas:

$${\rm{\Delta }}E1=\sqrt{{(LM3-LM1)}^{2}+{(aM3-aM1)}^{2}+{(bM3-bM1)}^{2}}$$

$${\rm{\Delta }}E2=\sqrt{{(LM3-LM2)}^{2}+{(aM3-aM2)}^{2}+{(bM3-bM2)}^{2}}$$

### Cell culture

Cytotoxicity was tested against pulp fibroblasts and human keratinocytes. Pulp fibroblasts were obtained from an intact third molar with incomplete root formation from one patient without systemic health problems. The patient was asked to donate the tooth for the study and, after the agreement, signed informed consent. Immortalized human keratinocyte cell line HaCaT were purchased (Banco de Células do Rio de Janeiro, Duque de Caxias, RJ, Brazil). Cells were cultured in DMEM complemented with 100 U/mL penicillin, 100 µg/mL streptomycin, 10% of FBS and 2 mM L-glutamine. Cells were cultured at 37 °C and under 5% CO_2_/95% air atmosphere. Every 3 days, cells received fresh medium. All reagents were purchased from Thermo Fisher Scientific (Waltham, Massachusetts, USA).

### SRB cytotoxicity assay

Eluates were prepared by immersing sealant disks measuring 3 mm in diameter and 1 mm in height (n = 3) into 1 mL of medium for 24 h at 37 °C. The cells were seeded in triplicate at a concentration of 5 × 10^3^ in 96 well plates prior to cell treatment. After 24 h, cells were treated with 100 µL of eluate. Controls were cultured without treatment. After 72 h, the cultures were fixed with 50 µl trichloroacetic acid 50% and stained with 50 μl SRB 0.4%. Quantification of viable cells was performed for absorbance at 560 nm in a microplate spectrophotometer (Multiskan™ GO, Thermo Fisher Scientific, USA) after suspension of cells in Trizma 10%. Wells without treatment were used as a control to normalize the percentage of viable cells.

### Mineral deposition

Mineral deposition was performed using micro-Raman spectroscopy (Senterra; Bruker Inc., Karlshure, Germany) equipped with a 100 mW diode laser, 785 nm wavelength and spectral resolution of ≈3.5 cm^−1^ with 3 co-additions during 5 s. Specimens discs measuring 4 mm × 2 mm were used. Baseline measurements were taken from sealant discs without treatment. One disc of each group was immersed in artificial saliva containing CaCl_2_·H_2_O, KH_2_PO_4_, KCl and NaCl^36^. After 7, 14 and 28 days of immersion, the phosphate peak of ~960 cm^−1^ was used to quantify the mineral deposition. A scanning electron microscope (SEM- JSM 6060, JEOL, Ltd., Tokyo, Japan) set to a voltage of 3 kV was used to evaluate the surface of specimens after 28 days of immersion in artificial saliva.

### Statistical analysis

Descriptive analysis was performed on specimens for micro-Raman and SEM images. The normality of data was evaluated using the Shapiro-Wilk test (p ≥ 0.05). The degree of conversion, the cytotoxicity and the contact angles of water and α-bromonaphthalene droplets over the specimens, the SFE and surface roughness data were analyzed using one-way ANOVA and Tukey’s test. The contact angles between resin sealants and enamel and colorimetry results were analyzed using two-way ANOVA and Tukey’s test. All tests were performed at 5% significance.

## Data Availability

The datasets generated during and/or analyzed during the current study are available from the corresponding author on reasonable request.

## References

[1] Wright, J. T. *et al*. Evidence-based Clinical Practice Guideline for the Use of Pit-and-Fissure Sealants. *Pediatr Dent* **38**, 120–136 (2016). 28206888

[2] Schwendicke F, Jäger AM, Paris S, Hsu LY, Tu YK (2015). Treating Pit-and-Fissure Caries: A Systematic Review and Network Meta-analysis. J Dent Res.

[3] Ahovuo-Saloranta A (2017). Pit and fissure sealants for preventing dental decay in permanent teeth. Cochrane Database Syst Rev.

[4] Mickenautsch S, Yengopal V (2016). Caries-Preventive Effect of High-Viscosity Glass Ionomer and Resin-Based Fissure Sealants on Permanent Teeth: A Systematic Review of Clinical Trials. PLoS ONE.

[5] Al-Jobair A, Al-Hammad N, Alsadhan S, Salama F (2017). Retention and caries-preventive effect of glass ionomer and resin-based sealants: An 18-month-randomized clinical trial. Dent. Mater. J..

[6] Urquhart, O. *et al*. Nonrestorative Treatments for Caries: Systematic Review and Network Meta-analysis. *J. Dent. Res*. 22034518800014, 10.1177/0022034518800014 (2018). 10.1177/0022034518800014 PMC6304695 30290130

[7] Schwendicke F, Stolpe M, Meyer-Lueckel H, Paris S (2015). Detecting and Treating Occlusal Caries Lesions. J Dent Res.

[8] Cheng L (2017). Developing a New Generation of Antimicrobial and Bioactive Dental Resins. J Dent Res.

[9] Muller-Bolla M (2018). Effectiveness of Resin-Based Sealants with and without Fluoride Placed in a High Caries Risk Population: Multicentric 2-Year Randomized Clinical Trial. Caries Res..

[10] Cury JA, de Oliveira BH, Dos Santos APP, Tenuta LMA (2016). Are fluoride releasing dental materials clinically effective on caries control?. Dent Mater.

[11] Chopra NG (1995). Boron nitride nanotubes. Science.

[12] Degrazia FW, Leitune VCB, Visioli F, Samuel SMW, Collares FM (2018). Long-term stability of dental adhesive incorporated by boron nitride nanotubes. Dent Mater.

[13] Genchi GG, Ciofani G (2015). Bioapplications of boron nitride nanotubes. Nanomedicine (Lond).

[14] Lahiri D, Singh V, Keshri AK, Seal S, Agarwal A (2011). Apatite formability of boron nitride nanotubes. Nanotechnology.

[15] Degrazia FW, Leitune VCB, Samuel SMW, Collares FM (2017). Boron nitride nanotubes as novel fillers for improving the properties of dental adhesives. J Dent.

[16] Besinis A, De Peralta T, Tredwin CJ, Handy RD (2015). Review of nanomaterials in dentistry: interactions with the oral microenvironment, clinical applications, hazards, and benefits. ACS Nano.

[17] Nautiyal, P. *et al*. Oxidative Unzipping and Transformation of High Aspect Ratio Boron Nitride Nanotubes into “White Graphene Oxide” Platelets. *Sci Rep* **6** (2016). 10.1038/srep29498 PMC4937397 27388704

[18] Tiano AL (2016). Thermodynamic approach to boron nitride nanotube solubility and dispersion. Nanoscale.

[19] Gao Z, Zhi C, Bando Y, Golberg D, Serizawa T (2011). Noncovalent functionalization of disentangled boron nitride nanotubes with flavin mononucleotides for strong and stable visible-light emission in aqueous solution. ACS Appl Mater Interfaces.

[20] Kim D (2014). Dispersion of boron nitride nanotubes in aqueous solution by simple aromatic molecules. J Nanosci Nanotechnol.

[21] Michelsen VB, Moe G, Strøm MB, Jensen E, Lygre H (2008). Quantitative analysis of TEGDMA and HEMA eluted into saliva from two dental composites by use of GC/MS and tailor-made internal standards. Dent Mater.

[22] Geurtsen W, Leyhausen G (2001). Concise Review Biomaterials & Bioengineering: Chemical-Biological Interactions of the Resin Monomer Triethyleneglycol-dimethacrylate (TEGDMA). Journal of Dental Research.

[23] Horváth L (2011). *In Vitro* Investigation of the Cellular Toxicity of Boron Nitride Nanotubes. ACS Nano.

[24] Ciofani G, Danti S, D’Alessandro D, Moscato S, Menciassi A (2010). Assessing cytotoxicity of boron nitride nanotubes: Interference with the MTT assay. Biochemical and Biophysical Research Communications.

[25] Zhang F, Xia Y, Xu L, Gu N (2008). Surface modification and microstructure of single-walled carbon nanotubes for dental resin-based composites. J. Biomed. Mater. Res. Part B Appl. Biomater..

[26] Douglas RD, Steinhauer TJ, Wee AG (2007). Intraoral determination of the tolerance of dentists for perceptibility and acceptability of shade mismatch. J Prosthet Dent.

[27] Premaraj TS (2014). An *in-vitro* evaluation of mechanical and esthetic properties of orthodontic sealants. Eur J Dent.

[28] Cagetti MG (2014). Effect of Fluoridated Sealants on Adjacent Tooth Surfaces. J Dent Res.

[29] Beun S, Bailly C, Devaux J, Leloup G (2008). Rheological properties of flowable resin composites and pit and fissure sealants. Dent Mater.

[30] Kühnisch J, Mansmann U, Heinrich-Weltzien R, Hickel R (2012). Longevity of materials for pit and fissure sealing–results from a meta-analysis. Dent Mater.

[31] Mickenautsch S, Yengopal V (2013). Validity of sealant retention as surrogate for caries prevention–a systematic review. PLoS ONE.

[32] Chen M, Yu Q, Sun H (2013). Novel Strategies for the Prevention and Treatment of Biofilm Related Infections. Int J Mol Sci.

[33] Liu Y, Zhao Q (2005). Influence of surface energy of modified surfaces on bacterial adhesion. Biophysical Chemistry.

[34] Park J, Song C, Jung J, Ahn S, Ferracane J (2012). The Effects of Surface Roughness of Composite Resin on Biofilm Formation of *Streptococcus mutans* in the Presence of Saliva. Operative Dentistry.

[35] Lahiri D (2011). Boron nitride nanotube reinforced hydroxyapatite composite: mechanical and tribological performance and *in-vitro* biocompatibility to osteoblasts. J Mech Behav Biomed Mater.

[36] Karlinsey RL, Hara AT, Yi K, Duhn CW (2006). Bioactivity of novel self-assembled crystalline Nb2O5 microstructures in simulated and human salivas. Biomed Mater.

[37] Featherstone JD, Fontana M, Wolff M (2018). Novel Anticaries and Remineralization Agents: Future Research Needs, Novel Anticaries and Remineralization Agents: Future Research Needs. J Dent Res.

[38] Kanzow P, Wiegand A, Schwendicke F (2016). Cost-effectiveness of repairing versus replacing composite or amalgam restorations. Journal of Dentistry.

[39] Collares FM (2014). Discrepancies in degree of conversion measurements by FTIR. Brazilian Oral Research.

[40] ten Cate JM, Duijsters PP (1982). Alternating demineralization and remineralization of artificial enamel lesions. Caries Res..

